# Management of Gartland Type 1 Supracondylar Fractures: A Systematic Review

**DOI:** 10.3389/fped.2022.863985

**Published:** 2022-05-19

**Authors:** Stephanie Coupal, Kenneth Lukas, Amy Plint, Maala Bhatt, Kevin Cheung, Kevin Smit, Sasha Carsen

**Affiliations:** ^1^Division of Orthopedic Surgery, Children’s Hospital of Eastern Ontario, University of Ottawa, Ottawa, ON, Canada; ^2^Division of Emergency Medicine, Children’s Hospital of Eastern Ontario, University of Ottawa, Ottawa, ON, Canada; ^3^Division of Plastic Surgery, Children’s Hospital of Eastern Ontario, University of Ottawa, Ottawa, ON, Canada

**Keywords:** pediatrics, systematic review, supracondylar humerus fractures, cast, splint

## Abstract

**Purpose:**

Gartland Type 1 supracondylar humerus fractures are stable, non-displaced injuries treated with non-operative management. This systematic review was performed to gather evidence on the optimal form of immobilization to treat these fractures.

**Methods:**

The review process was conducted according to the Preferred Reporting Items for Systematic Reviews and Meta-Analyses (PRISMA) guidelines. An electronic search was performed in November 2020. Articles were eligible if they included children less than 18 years old, with non-displaced supracondylar fractures, treated non-operatively. Randomized trials, quasi-experimental trials, and prospective cohort studies were included. Outcomes of interest included fracture displacement, pain control, time to return to normal activities, return of range of motion (ROM), child/parent satisfaction, adverse events, and cost. Risk of bias was assessed using the Newcastle-Ottawa scale, Rob-2, and the ROBINS tools.

**Results:**

After duplicate records were removed, 525 records were evaluated with 9 studies meeting the inclusion criteria and 5 reporting clinical outcomes. The studies were heterogenous, in intervention and outcomes, and all at moderate risk of bias. Within the available evidence there were no cases of fracture displacement. Two small studies suggested that cuff and collar treatment provided inadequate pain control and delay in return to normal activities, compared to posterior splints. Two randomized control trials (RCTs) suggested that soft fiberglass casts reduced appointment time and increased parent satisfaction, compared to traditional casts. No studies directly compared posterior splints to circumferential casts.

**Conclusion:**

There is insufficient high-quality evidence to determine the optimal conservative treatment for patients with Gartland type 1 supracondylar fractures. Level of Evidence Level II systematic review of Level II studies.

**Systematic Review Registration:**

[PROSPERO], identifier [CRD42020144616].

## Introduction

Supracondylar humerus fractures are the most common pediatric elbow fracture (1) and account for around 60–70 emergency department visits per 100,000 children annually (2). These fractures are categorized according to the modified Gartland classification system, depending on the degree of displacement, disruption of the posterior cortex, and location of the anterior humeral capitellum line on a lateral radiograph (3). Gartland Type I fractures are non-displaced and are widely accepted as stable fractures that should be treated non-operatively.

The ideal treatment for Type I supracondylar fractures should prevent fracture displacement and result in excellent clinical outcomes while minimizing adverse outcomes, pain, as well as direct and indirect costs to families and healthcare systems. Despite the common nature of these fractures, there remains a lack of consensus regarding which type of immobilization and follow-up care is most appropriate. Emergency department guidelines from Australia and Canada suggest immobilization with an above elbow “backslab” (a posterior splint) and broad arm sling. (4, 5). In comparison, long arm cast immobilization is generally recommended in a number of orthopedic surgery clinical guidelines and textbooks (6).

Given the frequency of the fracture and the existing clinical ambiguity with respect to the type of immobilization, a systematic review was performed to determine the optimal management of Type I supracondylar humerus fractures based on the highest level of evidence available. Our primary objective was to determine which forms of immobilization for Type I supracondylar humerus fractures prevent fracture displacement. Secondary objectives include the determination of relative risks and benefits of different treatment options.

## Materials and Methods

### Study Design

A systematic review was performed to identify publications that reported clinical outcomes and adverse events in pediatric patients with Type I supracondylar humerus fractures treated with immobilization. The review process was conducted according to the Preferred Reporting Items for Systematic Reviews and Meta-Analyses (PRISMA) guidelines, the details of which are available in Appendix A. The review protocol was published on PROSPERO (CRD42020144616).

### Search Strategy

The following databases were searched on November 13, 2020: MEDLINE, Embase, and CENTRAL Trials Registry of the Cochrane Collaboration, using the Ovid interface. Search terms for intervention included cast, slab, sling, cuff and collar, splint, non-surgical, and immobilization with the appropriate Boolean operators. Population-specific search terms included supracondylar fracture, or distal humerus fracture and babies, neonatal, infant, child, preschool, adolescent, or pediatric using corresponding Boolean operators. The search was not restricted by language or study design. Our search strategy was designed and conducted by a librarian experienced in systematic reviews, using a method designed to optimize term selection (7). A detailed description of the search strategies is presented in Appendix B.

### Study Selection

Studies were considered eligible if they met the following criteria: (1) the population included children < 18 years with type 1 (non-displaced) supracondylar fractures; (2) the study type was: randomized trial, quasi-experimental trial (non-randomized interventional study), or prospective cohort; (3) the study involved non-operative fracture management (including: tensor bandage, splint, casting, sling, cuff and collar, no intervention); (4) written in the English language. Fracture displacement was considered the primary outcome, but was not made an explicit inclusion criterion in order to broaden the article pool for reporting on our secondary outcomes. Studies were excluded if they met the following exclusion criteria: (1) narrative and systematic reviews, editorials, letters, surveys, case series and case reports, study protocols, retrospective cohort studies, cross-sectional studies, and studies published only in abstract form; (2) studies that primarily focused on closed reduction, operative management, or traction; (3) studies that primarily focused on adult patients; (3) animal studies; (4) studies that solely focused on patients with displaced (Gartland type II or III) supracondylar fractures, intra-articular distal humerus fractures, or proximal humerus fractures.

Duplicate records were removed, and records retrieved by the electronic search were uploaded to an online systematic review tool (InSight Scope, Ottawa, Canada). Records were appraised against the inclusion and exclusion criteria using a two-step approach. First, two reviewers (KL and SC) independently reviewed the titles and abstracts of the papers for potentially eligible studies. The full-text article of any abstract selected by either reviewer was then reviewed by both reviewers. Conflicts were resolved by the senior author (KL and SC). The reference list from the articles of the included studies was reviewed by KL and SC to identify any further possibly relevant articles. The authors of the included articles were contacted to inquire about additional available data or to clarify results or methodology if unclear.

### Data Extraction

Data from the included studies were extracted independently by two authors (KL and SC) and compared for consistency before inclusion in the analysis. Full data extraction included study design details, population, and outcomes including fracture displacement, pain control, time to return to normal activities, return of range of motion (ROM), child/parent satisfaction, adverse events, cost (health care, patient/parent, societal), and additional hospital visits. Discrepancies were investigated and rectified by returning to the original paper. In cases where the study population was heterogenous, data was extracted specifically for Type I or non-displaced fractures, where possible. There was no specific data manipulation required to extract this fracture-specific data.

### Quality Assessment

Risk of bias was assessed by two authors (KL and SC) with the Cochrane Rob-2 for randomized control trials (RCTs), Cochrane ROBINS-1 for quasi-experimental studies, and the Newcastle-Ottawa Scale for cohort studies. Discrepancies were resolved with discussion. There is low risk of selection or publication bias across this research topic.

### Data Synthesis

We had originally planned to perform a meta-analysis. However, given the heterogeneity of studies and results a descriptive analysis was instead performed.

## Results

### Study Selection

**FIGURE 1:**
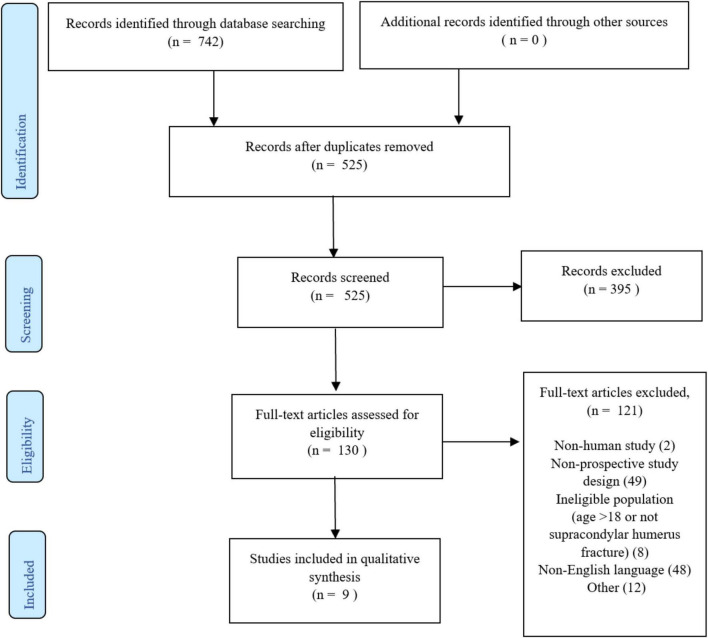
Prisma diagram.

The primary database search returned 742 records, and 525 records remained after duplicates were removed. Screening of titles and abstracts further excluded 367 records, leaving 126 for full article review with 9 studies meeting all the inclusion and exclusion criteria (see Figure 1). Four of the studies did not include clinical or adequate radiological outcome data, and therefore could not be used for data extraction. No additional studies were identified through review of the references of included papers.

### Study Characteristics and Methodological Quality

Studies which met the inclusion/exclusion criteria, and contained clinical data, are described in Table 1. There were 3 RCTs, 1 prospective cohort study, and 1 quasi-experimental study. Studies were performed in North America, Europe, and Australia and were all published in the last 20 years. Interventions investigated in the studies were posterior splint, long arm cast, cuff and collar, and “Blount’s immobilization” (cuff and collar with elbow at 100–120 degrees of flexion). Time of immobilization was inconsistently reported but varied from 2 weeks to 4 weeks. Follow-up time ranged from 2 days to 48 weeks. Details of the risk of bias assessment can be found in Tables 2, 3.

**TABLE 1:** Description of studies that met the inclusion criteria and included clinical outcome data.

Author, Year	Design	Location	Type of immobilization	Duration of immobilization	Outcomes reported	Age: Mean (range) in years	Number of participants
Leksan et al. (11)	Prospective Cohort	Europe	Humerus splint, cast, and Blount’s immobilization	Not reported	1. ROM	Not reported	38 (18 Type I)
Ballal et al. (7)	Quasi-experimental	Europe	A. Cuff and Collar B. Backslab	Not reported. Patients evaluated 2.67 days from injury (range 1-8 days) and then followed clinically	2. Fracture displacement (no formal radiographic follow-up) 3. Pain	A. 6 (2–14) B. 8 (4–14)	A. 20 B. 20
Oakley et al. (8)	RCT	Australia	A. Collar and Cuff B. Backslab and Sling	14–16 days, plus 2 additional weeks if tenderness/discomfort remained	1. Fracture displacement 2. Pain 3. ROM 4. Parent satisfaction 5. Time to return to normal activities 6. Costs (indirect)	A. 5.2 (2.9–6.9) B. 6.0 (4.6–8.1)	A. 23 B. 27 (Type I and elbow joint effusion with no visible fracture)
Silva et al. (9)	RCT	North America	A. Long Arm Cast (Traditional Hard Fiberglass) with sling B. Long Arm Cast (Soft fiberglass) with sling	4 weeks (8 week follow-up)	1. Fracture displacement 2. Pain 3. Parent satisfaction 4. ROM	A. 4.8 B. 5.4 Range not reported	A. 50 B. 50 (76% Type 1, other diagnosis include elbow effusion and other occult fracture)
Silva et al. (10)	RCT	North America	A. Long Arm Cast (Soft fiberglass) B Long Arm Cast (Soft fiberglass)	4 weeks (8 week follow-up)	1. Fracture displacement 2. Pain 3. ROM 4. Parent satisfaction 5. Costs (direct and indirect)	A. 5 (1.9 – 10.8) B. 5 (2.6 – 9.4)	A. 26 B. 26 (82% Type 1, other diagnosis include elbow effusion and other occult fracture)

**TABLE 2:** Risk of bias for prospective cohort studies - Newcastle–Ottawa score.

Study (Author et al., Year)	Selection (max 4 stars)	Comparability (max 2 stars)	Outcome (max 3 stars)
Leksan et al. (11)	0	0	*

**TABLE 3:** Risk of bias for randomized control trials (RCTs) (ROB-2) and non-RCTs (ROBINS-1).

Study	Experimental	Comparator	Primary outcome	Risk of bias tool used	Randomization process	Confounding	Selection of participipants	Classification of interventions	Deviations from intended interventions	Missing outcome data	Measurement of the outcome	Selection of the reported result/outcome	Overall
Oakley et al. (8)	Cuff and Collar	Backslab	Fracture displacement	ROB-2	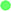	N/A	N/A	N/A	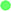	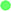	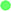	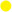	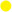 Some concern
Silva et al. (9)	Long arm cast (hard Fiberglass)	Long arm cast (soft Fiberglass)	Fracture displacement	ROB-2	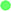	N/A	N/A	N/A	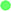	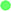	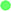	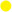	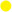 Some concern
Silva et al. (10)	Long arm cast (soft Fiberglass) removed at home	Long arm cast (soft Fiberglass) removed in office	Fracture displacement	ROB-2	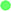	N/A	N/A	N/A	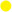	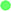	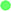	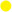	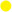 Some concern
Ballal et al. (7)	Backslab	Cuff and Collar	Fracture Displacement	ROBINS-1	N/A	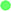	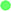	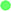	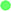	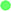	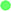	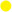	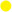 Some concern

*Legend: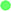 low risk of bias, 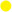 moderate risk/some concerns.*

### Outcomes Associated With Cuff and Collar and Posterior Splints

Two studies directly compared cuff and collar management to the use of a posterior splint (7, 8).

A quasi-experimental study by Ballal (7) included children who presented to a fracture clinic on an average of 2.7 days from injury. The patients had been treated with a cuff and collar or posterior splints based on the emergency physician preference. The posterior splints were placed with the elbow in “at least 90 degrees of flexion.” The authors report that “none of the fractures displaced during further management,” although there was no specific protocol for radiographic follow-up. The children treated with posterior splints had significantly less pain (3.4/10 vs. 7.2/10, *p* < 0.0001) and decreased regular analgesia use (4 times less, *p* = 0.0002) compared to those treated with cuff and collar. Furthermore, 85% of the patients treated with cuff and collar experienced sleep disturbance, compared to only 45% of the patients in the posterior splint group (7). Range of motion was not compared between the groups. There are moderate concerns with risk of bias since the study protocols were not published *a priori* (Table 3).

Oakley et al performed an RCT that investigated cuff and collar, compared to posterior splint (with the elbow placed at 90 degrees) (8). There were no cases of fracture displacement with either treatment, as measured on radiographs performed at the follow-up visit 12–16 days after injury. The primary outcome was the difference in pain intensity and duration and the parental or the patient’s willingness to use similar immobilization in the future. There was a trend toward decreased use of analgesia and duration of pain for the posterior splint compared to the cuff and collar (4 vs. 6 days, respectively), but statistical significance was not reported. Time to return to activity was also shorter in the posterior splint group (2 vs. 7 days, respectively). ROM restrictions was 50 degrees in the posterior splint group and 40 degrees in the cuff and collar group (*p* not reported). Differences in pain, analgesia use, and participation rates in usual activities had resolved by 4 weeks. Parent satisfaction, harms of immobilization, rates of parents who missed work, duration of missed work, and proportion of patients requiring days off from school/daycare demonstrated no differences between the methods of immobilization. Some concerns for risk of bias were identified for the Oakley study, as again there was no pre-specified, published protocol of outcomes prior to the commencement of the study (Table 3).

### Outcomes Associated With Long Arm Casts

Long arm casts were investigated in two randomized control studies by Silva et al. (9, 10). In 2018, the authors compared traditional fiberglass to soft fiberglass casts. Both types of casts were placed with the elbow at 90–100 degrees of flexion and the forearm in neutral rotation (9). This study reported no evidence of fracture displacement between the two groups. ROM and parent satisfaction were also found to be equivalent between groups at the 8 week follow-up appointment. Pain scores between the groups showed inconsistent results over time with no difference at 1 week, significant differences at 4 weeks, and no difference at 8 weeks. Overall risk of bias assessment showed some concerns due to measurement of outcome variables, since there was no mention of blinding of the radiographic assessors to intervention (Table 3).

In 2019, Silva et al investigated different methods of cast removal to improve parent satisfaction: clinic removal (Group A) was compared to removal at home by parents *via* telehealth appointment (Group B) (10). Soft fiberglass casts were used in both the groups studied. There were no cases of fracture displacement in either group. At latest follow-up there was no significant difference in the mean ROM, with Group A: 147 degrees and Group B: 151 degrees (*p* = 0.5). Significant difference in the length of appointment time was found between the groups, with Group A: 110.7 min and Group B: 17.6 min (*p* < 0.001). When the parents in the traditional clinic visit learned about the increased appointment time, their satisfaction dropped and was statistically lower than the telehealth group, which was 76.4% for Group A compared to 97.7% for Group B (*p* = 0.05). Despite this difference in appointment time there was no significant difference in mean professional fee [Group A: $29.22, Group B: $22.51(*p* = 0.19)]. Quality assessment showed some concern for risk of bias for this study due to deviations from the intended intervention; parents from both groups removed the cast prior to the intended date and the data from these patients was not included in the final analysis (Table 3). In addition, the radiographic assessors measuring the primary outcome were not blinded to intervention.

### Other Forms of Immobilization

Leksan et al. performed a prospective cohort study examining the functional status of the patient’s elbow after conservative treatment (11). They included 18 patients with Gartland type 1 fractures treated with a humerus splint, cast, or Blount’s immobilization. Results were not separated by the type of immobilization, but after completion of treatment, patients had an average ROM of 128.83 degrees, ranging from 110 to 140 degrees with a standard deviation of 8.65 degrees. No other clinical measures or outcomes were reported. This study is not of high quality, despite objective outcome measures. The patients selected were involved in traffic accidents (rollerblading or falling from a bicycle), which is a relatively high energy mechanism. In addition, it was unclear how the type of immobilization was ascertained, as there was no direct comparator, the length of follow-up was not described, and neither was loss to follow-up (see Table 2).

### Studies Without Clinical Outcome Reporting

Three prospective studies met the inclusion criteria but did not contribute any outcome data to our systematic review, as they did not include radiographic data and the clinical data was not reported specific to Type 1 fractures (i.e., it was grouped with Type 2 and Type 3 fractures treated operatively) (12–14). A small prospective study by Pudas on the utility of MRI in elbow fractures included patients with supracondylar humerus fractures but no clinical or radiographic follow-up data was reported (15).

## Discussion

### Fracture Displacement

The quality of evidence in these studies is low, and therefore we cannot make strong conclusions on the effect of each type of immobilization on fracture displacement. However, the results of this systematic review suggest that there is no fracture displacement with the use of cuff and collar, posterior splinting, or long arm casts (7–10). These findings are in alignment with other available literature including a retrospective review of 53 cases, which demonstrated that the use of posterior splint resulted in minimal changes in fracture displacement (16). Specifically, they found only 1 case of change in the anterior humeral line (from posterior 1/3 of the capitellum to middle 1/3) and 1 case of change in the humeral capitellum angle by 7 degrees, which is considered to be within the normal interrater measurement variability.

### Benefits of Immobilization

Cuff and collar immobilization appears to have fewer benefits for patients, compared to posterior splints. The use of cuff and collar resulted in a delayed return to normal activities (8), more interrupted sleep, and increased average pain scores especially early in the injury phase (7). In addition, the cuff and collar did not result in pain scores at levels considered to be the minimum for adequate pain control (<30 mm on a 100 mm visual analog scale), whereas the posterior splint achieved pain levels below this threshold (8). It is therefore reasonable to conclude that the results of this study suggest that immobilization with a splint or cast is significantly better for patients than cuff and collar alone. There were no consistent differences in pain scores between traditional fiberglass and soft cast. When comparing cuff and collar to posterior splints, there was no difference in parent satisfaction. Parent satisfaction for long arm casts was also reported by Silva, with a significant difference in parent satisfaction only when parents were informed of the increased appointment time associated with typical clinic appointments as compared to telehealth visits (9, 14).

Two studies investigated the possible benefits of the use of “soft cast,” otherwise known as “peelable fiberglass” casts, utilizing a form of fiberglass which can be removed at the end of a period of immobilization by a parent at home (9, 10). Such “soft cast” has been investigated for immobilization of pediatric buckle fractures (17), and in small studies appear to result in high patient/parent satisfaction (18). The studies included in this review showed a similar improvement in parental satisfaction. However, these studies contained small patient numbers, and patients who removed their cast prematurely at home were excluded from the final analysis. Therefore, the effect of non-compliance during soft cast treatment of supracondylar humerus fractures is unknown. In addition, it is unclear whether “soft cast” treatment option is widely known, available, or considered reasonable to the pediatric emergency medicine and pediatric orthopedic surgery community. For these reasons, future investigation into this treatment method is warranted.

### Harms of Immobilization

Orthopedic surgery visits have previously been reported to result in direct costs and societal costs due to loss of productivity for parents (19). Direct healthcare costs were only reported in one study of long arm casts, with no significant difference in cost despite an increased length of appointment in the traditional fiberglass cast (9). The type of immobilization directly impacts the follow-up required, and therefore has downstream societal costs. Rates of missed parental working days, duration of missed work, and proportion of patients requiring days off from school/daycare were only compared between cuff and collar and posterior splint in only one study, but no differences were reported (8).

High rates of improperly placed extremity splints have been reported in other literature, and associated with skin and soft tissue complications (20). However, direct physical harms of immobilization (or lack thereof) were not reported in any of the studies included in this review.

### Additional Management Considerations

The ideal length of time required to immobilize Type I supracondylar humerus fractures is not clear from the evidence gathered in this review. In addition, an appropriate type of clinical follow-up care is not well defined. Telehealth has been advocated for delivering orthopedic clinical care during the COVID-19 pandemic (21), and is often used in rural/suburban areas, where it can greatly reduce the appointment times in the form of waiting and travel time (22). One small RCT compared telehealth clinical visits to traditional visits, and more research is warranted prior to changing the clinical follow-up methods.

### Limitations

This systematic review is limited in its conclusions by the heterogeneity of the individual studies, which investigated multiple forms of immobilization. There is no study focused on comparing posterior splints and casts, making it impossible to directly compare these two common treatments. The majority of studies also included patients with a variety of elbow injuries (such as occult elbow injuries), which may be more inherently stable than Type I injuries with a visible fracture line. Not all studies included a description of formal radiographic follow-up (8), which restricts the reliability of fracture displacement reporting. In addition, scarce information was presented on adverse events, and therefore it is unclear if these were not present or simply not recorded. A major limitation of the evidence is the short, formal follow-up period (average of 2.7 days to 8 weeks), meaning that long-term ROM and functional data are not available.

## Conclusion

Despite the ubiquity of the fracture, there remains very limited high-quality evidence on the treatment of Type 1 supracondylar humerus fractures. In addition, there is significant heterogeneity in the intervention and outcome measures in the current literature. Based on the best available evidence, Type 1 supracondylar fractures are stable fractures with no evidence of displacement reported, regardless of the form of immobilization used. Posterior splint and circumferential long arm casts both provide adequate pain control and early return to activity, whereas cuff and collar alone has been shown to be comparatively less effective or even inadequate for pain control. Therefore, immobilization with a splint or cast is reasonable to recommend. It was not possible to determine the optimal duration and type of immobilization for these fractures. Interestingly, soft fiberglass casts may offer the potential of rigid immobilization with the option of cast removal at home, which, in a small study, resulted in reduced appointment times and increased patient/parent satisfaction while maintaining fracture stability. The results of this systematic review clarify the limitations of the existing evidence, and may help to serve as a guide toward the development of more definitive evidence and guidelines. Further research is needed to better determine the optimal management of Type 1 supracondylar humerus fractures in children.

## Data Availability Statement

The raw data supporting the conclusions of this article will be made available by the authors, without undue reservation.

## Author Contributions

SCo: study design, data capture, measurements, analysis, and manuscript preparation and revision. KL: data capture, measurements, and analysis. AP and MB: study design, analysis, and manuscript preparation and revision. KC: analysis and manuscript preparation and revision. KS: study design and manuscript preparation and revision. SCa: study design, measurements, analysis, and manuscript preparation and revision. All authors contributed to the article and approved the submitted version.

## Conflict of Interest

The authors declare that the research was conducted in the absence of any commercial or financial relationships that could be construed as a potential conflict of interest.

## Publisher’s Note

All claims expressed in this article are solely those of the authors and do not necessarily represent those of their affiliated organizations, or those of the publisher, the editors and the reviewers. Any product that may be evaluated in this article, or claim that may be made by its manufacturer, is not guaranteed or endorsed by the publisher.

## Appendix A

**Table:**

Section/topic	#	Checklist item	Reported on page #
**TITLE**			
Title	1	Identify the report as a systematic review, meta-analysis, or both.	Title Page
**ABSTRACT**			
Structured summary	2	Provide a structured summary including, as applicable: background; objectives; data sources; study eligibility criteria, participants, and interventions; study appraisal and synthesis methods; results; limitations; conclusions and implications of key findings; systematic review registration number.	1–2
**INTRODUCTION**			
Rationale	3	Describe the rationale for the review in the context of what is already known.	3
Objectives	4	Provide an explicit statement of questions being addressed with reference to participants, interventions, comparisons, outcomes, and study design (PICOS).	3
**METHODS**			
Protocol and registration	5	Indicate if a review protocol exists, if and where it can be accessed (e.g., Web address), and, if available, provide registration information including registration number.	4
Eligibility criteria	6	Specify study characteristics (e.g., PICOS, length of follow-up) and report characteristics (e.g., years considered, language, publication status) used as criteria for eligibility, giving rationale.	4
Information sources	7	Describe all information sources (e.g., databases with dates of coverage, contact with study authors to identify additional studies) in the search and date last searched.	4
Search	8	Present full electronic search strategy for at least one database, including any limits used, such that it could be repeated.	Appendix
Study selection	9	State the process for selecting studies (i.e., screening, eligibility, included in systematic review, and, if applicable, included in the meta-analysis).	4–5
Data collection process	10	Describe method of data extraction from reports (e.g., piloted forms, independently, in duplicate) and any processes for obtaining and confirming data from investigators.	5–6
Data items	11	List and define all variables for which data were sought (e.g., PICOS, funding sources) and any assumptions and simplifications made.	5–6
Risk of bias in individual studies	12	Describe methods used for assessing risk of bias of individual studies (including specification of whether this was done at the study or outcome level), and how this information is to be used in any data synthesis.	6 (Table 2–3)
Summary measures	13	State the principal summary measures (e.g., risk ratio, difference in means).	N/A (descriptive analysis only due to heterogenous studies)
Synthesis of results	14	Describe the methods of handling data and combining results of studies, if done, including measures of consistency (e.g., I^2^) for each meta-analysis.	N/A
Risk of bias across studies	15	Specify any assessment of risk of bias that may affect the cumulative evidence (e.g., publication bias, selective reporting within studies).	6
Additional analyses	16	Describe methods of additional analyses (e.g., sensitivity or subgroup analyses, meta-regression), if done, indicating which were pre-specified.	N/A
**RESULTS**			
Study selection	17	Give numbers of studies screened, assessed for eligibility, and included in the review, with reasons for exclusions at each stage, ideally with a flow diagram.	Figure 1
Study characteristics	18	For each study, present characteristics for which data were extracted (e.g., study size, PICOS, follow-up period) and provide the citations.	Table 1
Risk of bias within studies	19	Present data on risk of bias of each study and, if available, any outcome level assessment (see item 12).	Table 2/3
Results of individual studies	20	For all outcomes considered (benefits or harms), present, for each study: (a) simple summary data for each intervention group (b) effect estimates and confidence intervals, ideally with a forest plot.	a) 7–11 b) N/A
Synthesis of results	21	Present results of each meta-analysis done, including confidence intervals and measures of consistency.	N/A
Risk of bias across studies	22	Present results of any assessment of risk of bias across studies (see Item 15).	6 (N/A)
Additional analysis	23	Give results of additional analyses, if done (e.g., sensitivity or subgroup analyses, meta-regression [see Item 16]).	N/A
**DISCUSSION**			
Summary of evidence	24	Summarize the main findings including the strength of evidence for each main outcome; consider their relevance to key groups (e.g., healthcare providers, users, and policy makers).	12–15
Limitations	25	Discuss limitations at study and outcome level (e.g., risk of bias), and at review-level (e.g., incomplete retrieval of identified research, reporting bias).	14–15
Conclusions	26	Provide a general interpretation of the results in the context of other evidence, and implications for future research.	15
**FUNDING**			
Funding	27	Describe sources of funding for the systematic review and other support (e.g., supply of data); role of funders for the systematic review.	N/A

*From: Moher D, Liberati A, Tetzlaff J, Altman DG, The PRISMA Group (2009). Preferred Reporting Items for Systematic Reviews and Meta-Analyses: The PRISMA Statement. PLoS Med 6(7): e1000097. doi: 10.1371/journal.pmed1000097.*

*For more information, visit: www.prisma-statement.org.*

## Appendix B

Searches were conducted using an Ovid multi-database search, and duplicate records were removed online giving preference to MEDLINE, then Embase, with no field preference. Lines 1–4 are optimized for MEDLINE and the main question constructs are broken out in separate lines for clarity. Lines 5–11 are optimized for Embase and lines 12–15 are optimized for CENTRAL. The next lines associated the records to the database the search was designed for, combine those sets and then remove duplicate records and finally isolate the records from each database again so each can be downloaded and imported into the citation manager using a database-specific import filter.

Database specific: MEDLINE including Epub Ahead of Print, In-Process and Other Non-Indexed Citations (1946- November 13, 2020) and Embase (1947- 2020, November 13) and the CENTRAL Trials Registry of the Cochrane Collaboration (October 20 issue) using the Ovid interface.

1. [(Supracondyl* adj2 fracture*) or gartland].ti,ab,kf.
2. Casts, Surgical/or Splits/or [conservative or cast* or immobili* or backslab* or slab* or sling* or (cuff* adj2 collar*) or splint* or non-surg*].ti,ab,kf. or “Referral and Consultation”/
3. (child* or adolescent* or infan*).mp.
4. (1 and 2 and 3) not (femur* or femoral or distal radi* or displaced or type ii or type 11 or type 2 or type two or type 3 or type 111 or type iii or type three).ti.
5. gartland type i supracondylar humerus fracture/or humerus supracondylar fracture/or humeral supracondylar fracture/or distal humeral fracture/or (distal humerus/and fracture*.mp.)
6. [(Supracondyl* adj2 fracture*) or gartland].ti,ab,kw. not (femur* or femoral or distal radi*).ti.
7. 5 or 6
8. exp “casts and noninvasive traction devices”/ or exp splint/or [conservative or cast* or Immobili* or backslab* or slab* or sling* or (cuff* adj2 collar*) or splint* or non-surg*].ti,ab,kw. or patient referral/
9. (baby or babies or newborn* or infan* or neonat* or preschool* or pre-school* or child* or pediatr* or paediatr* or teen* or adolescen*).mp.
10. (7 and 8 and 9) not (femur* or femoral or distal radi* or displaced or type ii or type 11 or type 2 or type two or type iii or type 111 or type 3 or type three).ti.
11. limit 10 to embase
12. [(Supracondyl* adj2 fracture*) or gartland].ti,ab,kw. not (femur* or femoral or distal radi*).ti.
13. [conservative or cast* or Immobili* or backslab* or slab* or sling* or (cuff* adj2 collar*) or splint* or non-surg*].ti,ab,kw.
14. (baby or babies or newborn* or infan* or neonat* or preschool* or pre-school* or child* or pediatr* or paediatr* or teen* or adolescen*).mp.
15. (12 and 13 and 14) not (femur* or femoral or distal radi* or displaced or type ii or type 11 or type 2 or type two or type iii or type 111 or type 3).ti.
16. 4 use medall
17. 11 use emczd
18. 15 use cctr
19. or/16-18
20. 19 use medal
21. 19 use emczd
22 19 use cctr
1. [(Supracondyl* adj2 fracture*) or gartland].ti,ab,kf.
2. Casts, Surgical/or Splits/or [conservative or cast* or immobili* or backslab* or slab* or sling* or (cuff* adj2 collar*) or splint* or non-surg*].ti,ab,kf. or “Referral and Consultation”/
3. (child* or adolescent* or infan*).mp.
4. (1 and 2 and 3) not (femur* or femoral or distal radi* or displaced or type ii or type 11 or type 2 or type two or type 3 or type 111 or type iii or type three).ti.
5. gartland type i supracondylar humerus fracture/or humerus supracondylar fracture/or humeral supracondylar fracture/or distal humeral fracture/or (distal humerus/and fracture*.mp.)
6. [(Supracondyl* adj2 fracture*) or gartland].ti,ab,kw. not (femur* or femoral or distal radi*).ti.
7. 5 or 6
8. exp “casts and noninvasive traction devices”/or exp splint/or [conservative or cast* or Immobili* or backslab* or slab* or sling* or (cuff* adj2 collar*) or splint* or non-surg*].ti,ab,kw. or patient referral/
9. (baby or babies or newborn* or infan* or neonat* or preschool* or pre-school* or child* or pediatr* or paediatr* or teen* or adolescen*).mp.
10. (7 and 8 and 9) not (femur* or femoral or distal radi* or displaced or type ii or type 11 or type 2 or type two or type iii or type 111 or type 3 or type three).ti.
11. limit 10 to embase
12. [(Supracondyl* adj2 fracture*) or gartland].ti,ab,kw. not (femur* or femoral or distal radi*).ti.
13. [conservative or cast* or Immobili* or backslab* or slab* or sling* or (cuff* adj2 collar*) or splint* or non-surg*].ti,ab,kw.
14. (baby or babies or newborn* or infan* or neonat* or preschool* or pre-school* or child* or pediatr* or paediatr* or teen* or adolescen*).mp.
15. (12 and 13 and 14) not (femur* or femoral or distal radi* or displaced or type ii or type 11 or type 2 or type two or type iii or type 111 or type 3).ti.
16. 4 use medall
17. 11 use emczd
18. 15 use cctr
19. or/16-18
20. 19 use medal
21. 19 use emczd
22. 19 use cctr
